# Surface Modification of 3D-Printed PCL/BG Composite Scaffolds via Mussel-Inspired Polydopamine and Effective Antibacterial Coatings for Biomedical Applications

**DOI:** 10.3390/ma15238289

**Published:** 2022-11-22

**Authors:** Kanwal Ilyas, Muhammad Asim Akhtar, Ezzeddine Ben Ammar, Aldo R. Boccaccini

**Affiliations:** Department of Materials Science and Engineering, Institute of Biomaterials, University of Erlangen-Nuremberg, Cauerstr. 6, 91058 Erlangen, Germany

**Keywords:** 3D printing, FDM, scaffolds, surface modification, bioactive glass, composite coating

## Abstract

A wide variety of composite scaffolds with unique geometry, porosity and pore size can be fabricated with versatile 3D printing techniques. In this work, we fabricated 3D-printed composite scaffolds of polycaprolactone (PCL) incorporating bioactive glass (BG) particles (13-93 and 13-93B3 compositions) by using fused deposition modeling (FDM). The scaffolds were modified with a “mussel-inspired surface coating” to regulate biological properties. The chemical and surface properties of scaffolds were analyzed by Fourier transform infrared spectroscopy (FTIR), contact angle and scanning electron microscopy (SEM). Polydopamine (PDA) surface-modified composite scaffolds exhibited attractive properties. Firstly, after the surface modification, the adhesion of a composite coating based on gelatin incorporated with strontium-doped mesoporous bioactive glass (Sr-MBGNs/gelatin) was significantly improved. In addition, cell attachment and differentiation were promoted, and the antibacterial properties of the scaffolds were increased. Moreover, the bioactivity of these scaffolds was also significantly influenced: a hydroxyapatite layer formed on the scaffold surface after 3 days of immersion in SBF. Our results suggest that the promoting effect of PDA coating on PCL-BG scaffolds leads to improved scaffolds for bone tissue engineering.

## 1. Introduction

Human bones are known for their self-healing ability, and it has been proven that they can self-regenerate and remodel without scarring [1]. However, in case of large bone defects that may result from infection, accidental damage or tumor resection, therapeutic intervention to stimulate the process of bone repair and regeneration is required [2,3]. Treatment via autologous bone grafting is considered the gold standard strategy to overcome large bone defects. However, bone grafting from some other part of the human body into the defect site demands further surgery, which may increase the probability of infection, trauma and morbidity [4,5]. These days, additive manufacturing (AM) is being highlighted as a game-changing technology in several tissue engineering applications [6]. For example, with the assistance of 3D printing methods, tailor-made porous scaffolds with superior pore morphology, controlled pore size and porosity can be fabricated [6]. Generally, in traditional scaffold processing methods, for example, the foam replica method, porogen templating, freeze-drying and solvent casting, it is almost impossible to control with high precision the interconnectivity, pore size and porosity of 3D scaffolds [7]. Therefore AM, as an advanced approach to design and manufacture 3D structures, is replacing the need for autografts and allografts [8,9]. Through computer-aided design (CAD) software and AM, complex 3D-printed scaffolds with controlled pore size can be successfully fabricated [8,10,11]. As a result, the regeneration of any specific damaged tissue or bone defect could be achieved without the need for bone transplantation [12]. Indeed, bone remodeling can be improved with the help of additively manufactured scaffolds, which offer a suitable environment for cells, providing a temporary matrix to support or direct cell differentiation and ultimately deliver a better mechano-induction process [13]. The AM of polymer-based scaffolds specially for bone reconstruction can be achieved by various techniques, for example, stereolithography, selective laser melting/sintering, and solution printing [8,9,11,14]; one of them is fused deposition modeling (FDM), a solvent-free approach useful to fabricate polymer-based composite scaffolds [8,11]. The printing of scaffolds using FDM is primarily dependent on the availability of pre-fabricated composite filaments, which are later processed in a printing process through an extrusion nozzle (at suitable temperature) to fabricate 3D scaffolds without using solvents [8,11].

With the advancement of technologies for the fabrication of novel 3D-printed scaffolds, PCL-based scaffolds have been attracting the attention of scientists because of the low melting temperature of PCL of approximately 60 °C. PCL is thus very convenient to fabricate 3D-printed structures by FDM [15]. On the other hand, PCL is inherently hydrophobic; therefore, it lacks the essential, required bioactivity [16]. To overcome these drawbacks, the fabrication of composite scaffolds has been frequently reported [17]. Hutmacher [11] reported the first successful fabrication of PCL/hydroxyapatite composite scaffold via FDM. These days, composite scaffolds with bioactive glass are well-known because of the superior osteoinductive and osteoconductive properties provided by bioactive glass (BG) [18,19]. Kolan et al. [15], for example, prepared a PCL/borate BG composite by dispersing BG particles into a chloroform-PCL solution to form a printable paste; later, the authors fabricated 3D PCL/borate BG composite scaffolds through pressure-based extrusion. Distler et al. [20] recently reported the fabrication of PLA-BG composite scaffolds through FDM; the authors observed an increase in bioactivity and biocompatibility with higher BG content. Moreover, the authors also reported that the complete encapsulation of BG particles in the polymer hindered the release of ions, which affected the biological properties of the scaffolds. Poh et al. [21] suggested that biological properties of a polymer-based composite scaffold can be enhanced by coating the surface with suitable biomaterials. However, the hydrophobicity of polymers usually hinders the adhesion of the coating layers [21]. To improve the surface properties of biomaterials, a unique method of surface modification known as “mussel-inspired polydopamine” has been put forward for biomedical applications [22,23]. The phenomenon originated from the mussel’s attachment to rocks in an aqueous environment. It was observed that mussels secret special proteins largely based on lysine and dihydroxyphenylalanine (DOPA) that cause their adhesion to rocks [23]. Interestingly, dopamine (DA) also has a similar amine functional group and catechol functional group that is present in the side chain of dihydroxyphenylalanine residues. One of the unique properties of polydopamine is its self-polymerization ability, which causes the oxidation of dopamine in a weak buffer solution. Therefore, it can easily accumulate on different hydrophobic and hydrophilic surfaces [24]. This base-activated polymerization and oxidation of polydopamine also facilitates the rapid and smooth coating of different materials onto the surface of hydrophobic scaffolds [25], because the hydrophilicity of surfaces modified with polydopamine (PDA) acts as a platform for effective adhesion of other biomaterials. Moreover, PDA can covalently immobilize different adhesive proteins [25]. Another effect of PDA modification is to improve the interaction with bioactive functional groups by self-assembled PDA and/or composite nano-layer formation; therefore, superior cell attachment and proliferation can be expected [26].

Taking inspiration from this surface modification strategy, in the present study, a simple and unique protocol for PDA-assisted coating on 3D-printed PCL composite scaffolds was introduced. Initially PCL/BG composite scaffolds were fabricated using silicate (1393 BG) or borate (1393B3 BG) particles as filler in PCL. The composite scaffolds were then coated with Sr-doped mesoporous bioactive glass nanoparticles (Sr-MBGNs) and gelatin before and after surface modification with PDA. The aim of this study was thus to enhance the bioactivity of PCL-based scaffolds in order to provide a suitable environment for cellular growth and proliferation and to overcome microbial infections. The outcomes of this project suggest that the successful composite coating via PDA surface modification leads to bioactive scaffolds with suitable properties and application potential in bone tissue engineering.

## 2. Materials and Methods

### 2.1. Materials

For scaffold fabrication, PCL was purchased from Sigma Aldrich, Taufkirchen, Germany (average MW 80,000 Da). Furthermore, dopamine hydrochloride (MW 189.4 g/mol) from Sigma-Aldrich (St. Louis, MO, USA), porcine gelatin type A from Sigma-Aldrich (St. Louis, MO, USA) and genipin (molar mass: 226.226 g/mol) from Wako (Osaka, Japan) were used for developing the coatings.

### 2.2. Fabrication of PCL-Bioactive Glass (BG) Filaments

Firstly, composite filaments of PCL and BG were prepared. For this purpose, two different BGs, namely a silicate (13-93) and a borate (13-93B3) glass (synthesized via melt quenching method and with particle size in the range 4–20 µm [27]), were used. The BG compositions are given in Table 1. The first step of composite filament fabrication was to mix PCL with BG particles. In order to achieve a homogeneous BG particle distribution in the polymer matrix, the chemical mixing was performed by using chloroform as a solvent to dissolve PCL (1:1 ratio). PCL was weighed and put into a beaker on a hot plate set to 50 °C. Chloroform was then added to the beaker to fully dissolve PCL. The BG powder was added slowly into the PCL-chloroform solution while stirring to obtain a homogenous distribution of BG particles in the PCL matrix. After visual confirmation that no agglomeration of BG particles could be found in the solution, the mixture was left for drying under a fume hood for 2 h. The dried PCL-BG mixture (composite) was then cut into small pieces and rolled into small pellets by hand. The pellets were then left for drying under a fume hood for 24 h. The prepared composite pellets were poured into the hopper of a filament extruder (NEXT 2.0, 3Devo B.V., Utrecht, The Netherlands). PCL-BG composite material was carefully fed in small quantities in order to decrease the risk of material agglomeration. To ensure a constant supply of material, the extrusion screw should be constantly filled with material (PCL-BG). When the diameter of a filament reached a value ≥1 mm, then the cooling fans of the extruder were immediately turned on. The filament was carefully collected onto a spool after a stable target diameter of 2.85 mm was attained. Filaments with a nominal diameter of 2.85 ± 0.15 mm were acceptable for final scaffold printing. After the production of each filament, the extruder was purged with a high-molecular-weight polyethylene (HMWPE, 3Devo B.V., Utrecht, The Netherlands) cleaning polymer. For the production of suitable filaments, heating parameters were adjusted to 120, 130, 130, and 140 °C from heaters 4 to 1, respectively. Moreover, the speed of the screw was set to approximately 5–6 rpm with the maximum fan speed.

### 2.3. Fabrication of Scaffolds

After the filament preparation, cylindrical scaffolds (10 mm diameter, 3 mm height) with interconnected pores (800 µm pore size) were fabricated by using a 3D printer (FDM, Ultimaker S5 Premium, Ultimaker B.V., Utrecht, The Netherlands). Scaffolds were designed with the help of computer-aided design (CAD) by using both Solid Edge (Siemens AG, Munich, Germany) and browser-based CAD tool TINKERCAD (Autodesk Inc., San Rafael, CA, USA). After that, the designed model was sliced by a supporting slicing software (Ultimaker Cura, Ultimaker B.V., Utrecht, The Netherlands) and finally, scaffolds were fabricated by 3D printing.

### 2.4. Sr-MBGNs/Gelatin Coating onto Mussel-Inspired PDA-Modified Scaffolds

The surface modification of scaffolds with PDA was achieved via direct immersion of scaffolds into a PDA buffer solution. Before PDA modification, the scaffolds were first rinsed with deionized water and then immersed into 0.5 mL of polydopamine buffer solution (prepared by dissolving 2 mg/mL dopamine hydrochloride (Sigma-Aldrich, St. Louis, MO, USA) in 10 mM Tris, pH 8.5). The immersed scaffolds were then placed in an incubator at room temperature and at a speed of 25 rpm. After 2 h, scaffolds were retrieved, rinsed several times with deionized water and dried in an incubator with a sufficient supply of nitrogen [16]. After that, a coating of strontium-doped mesoporous bioactive glass nanoparticles (Sr-MBGNs) and porcine gelatin (type A, Sigma-Aldrich, St. Louis, MO, USA) was prepared by following the previously published protocol [28]. Briefly, gelatin (5 wt%) was dissolved in deionized water at 50 °C and stirred for 30 min. After that, 20 wt% Sr-MBGNs (prepared via sol-gel method [28]) were added to the gelatin solution. A well-known cross-linker, genipin (1% wt/v) (Wako, Osaka, Japan), was then dissolved in ethanol and added to the rest of the solution (genipin/gelatin 1 wt%) while stirring for 30 min at 50 °C. Genipin is a cross-linker that is used to enhance mechanical properties of gelatin; however, a high concentration of genipin can lead to toxicity of scaffolds, affecting biocompatibility and cell regeneration [29]. The solution was then used for surface coating of the PCL-based 3D-printed scaffolds. For coating of scaffolds, the dip-coating technique was used; the scaffolds were soaked in the coating solution for 3 to 5 min, retrieved on tissue paper and then left to dry at room temperature in a desiccator with N_2_ environment for 24 h. Scaffolds coated without PDA modification were named “coating 1”, whereas “coating 2” referred to the coated scaffolds after PDA modification.

### 2.5. Characterization

#### 2.5.1. Surface Morphology, Porosity and BG Distribution

High-resolution imaging of scaffolds and coatings was obtained by scanning electron microscopy (Carl Zeiss™ AG, Jena, Germany). To avoid the charging of the samples, gold sputtering was performed prior to the analysis. Furthermore, the scaffold’s strut, pore size and structure were analyzed with an optical microscope (Stemi 508, Carl Zeiss, Jena, Germany). Further processing of images was performed with the help of ImageJ software.

The porosity of scaffolds was assessed by first measuring the weight of scaffolds before and after coating to calculate the weight of the coating material on the scaffold surface. The scaffold porosity after coating was calculated using the formula:

(1) $$P=(1-\frac{\rho_{s}}{\rho_{b}}-\frac{\rho_{cm}}{\rho_{ct}})\times 100$$

where *ρ_s_* is the density of the scaffold before coating, *ρ_b_* is the density of bioactive glass given in the literature [30], *ρ_cm_* is the coating density, and *ρ_ct_* is the theoretical density of the coating material. *ρ_cm_* was calculated for each sample as:

(2) $$\rho_{cm}=\frac{W_{ac}-W_{bc}}{V_{s}}$$

where *W_bc_* and *W_ac_* are respectively the weight of scaffold before and after coating. *V_S_* is the volume of the scaffold. The theoretical density of coating material *ρ_ct_* is estimated by the mixture rule as:

*ρ_ct_* = *X*_Gel_
*ρ*_Gel_ + *X_MBGNs_ρ_MBGNs_* (3)

where *X* represents the mass fractions of material in the coating, and *p* is its density of the coating components (gelatin and MBGNs).

#### 2.5.2. Contact Angle Measurement

Drop Shape Analysis System (Krüss, DSA30, Krüss GmbH, Hamburg, Germany) was used for the wettability assessment of the samples. A series of small squares (n = 3) of size 7 mm × 7 mm × 0.5 mm were printed to determine the wettability of different materials.

#### 2.5.3. In Vitro Degradation

For the evaluation of degradation, the initial weights of all 3D-printed scaffold were measured, and pictures were taken with a light microscope before immersion into the growth medium. The in vitro degradation study of PCL and PCL/BG composite scaffolds was carried out by soaking a set of 3 scaffolds per group into 5 mL of Dulbecco’s phosphate-buffered saline (DPBS) (pH 7.4, Gibco^®^, life technologies TM, Darmstadt, Germany) in a 25 mL cell culture tube (SARSTEDT AG & Co. KG, Nümbrecht, Germany). The tubes were placed inside a shaker with an integrated incubator (Heidolph Unimax 1010, Heidolph Instruments GmbH & CO. KG, Schwabach, Germany) at a temperature of 37 °C. The degradation of scaffolds was evaluated after 1, 3, 6 and 9 days. The scaffolds were taken out after the given days of immersion and placed on tissue paper to extract the excess solution. The samples were dried overnight. The weight of the coated scaffold after degradation and drying was measured. The weight loss percentage of coating material on the scaffold surface was calculated by the formula:

(4) $$Coat\;weight\;loss\left(\%\right)=\frac{W_{ac-}W_{c-dry}}{W_{ac}-W_{bc}}\times 100\%$$

where, *W_ac_* is weight of scaffold after coating, *W*_*c*-*dry*_ represents the dried weight of coated scaffold after degradation and *W_bc_* is the weight of scaffold before coating.

#### 2.5.4. Mechanical Properties

The mechanical strength of 3D-printed scaffolds was evaluated in compression by using a universal testing machine (Instron 3300 Floor Model, Instron^®^ GmbH, Darmstadt, Germany). The test was conducted according to the ASTM D695 standard, at a speed of 1.3 mm/min. The initial distance was set very close to the height of the 3D-printed scaffolds, while the absolute displacement for compression was set at 3 mm. The surface area of the scaffolds was determined before the measurement to determine the Young’s modulus. For each group of scaffolds (n = 3), closeup images were captured with an optical microscope (ZEISS Stemi 508, Zeiss AG, Ostfildern, Germany), and the surface area of scaffolds was calculated with ImageJ software.

#### 2.5.5. Bioactivity Study in Simulated Body Fluid

Simulated body fluid (SBF) was prepared as described by Kokubo et al. [31] to mimic the ion concentrations of human blood plasma. A set of n = 6 prismatic samples with size 7 mm × 7 mm × 0.5 mm were printed for each composite group. The required amount of SBF solution was calculated using the following formula:

(5) $$V_{s}=S_{a}/10$$

whereas the volume of SBF (*V_s_*) is in mL and the surface area (*S_a_*) of the square sample in mm^2^. The scaffolds were immersed in SBF solution and placed in an incubator (Heidolph Unimax 1010, Heidolph Instruments GmbH & CO. KG, Schwabach, Germany) at 37 °C. The SBF solution was changed every 2 days. The incubation times of 7, 14 and 28 days were chosen for non-coated scaffolds, whereas for the coated samples, 3 and 7 days were selected. After incubation for the specific time intervals, the samples were characterized with Fourier transform infrared spectroscopy (FTIR) (Shimadzu IRAffinity-1S, Shimadzu Corp, Tokyo, Japan). All spectra were obtained in absorbance mode and in the range of 4000 to 400 cm^−1^. Moreover, samples were also evaluated by scanning electron microscopy (SEM) and energy dispersed X-ray spectroscopy (EDX) at 20 KV.

#### 2.5.6. Antibacterial Study

To evaluate the antibacterial activity of scaffolds, two different bacterial strains were used, namely *Escherichia coli* (*E. coli*) and *Staphylococcus aureus* (*S. aureus*), Gram-negative and Gram-positive, respectively. The scaffolds were first sterilized under UV light for about 2 h. The colonies of bacteria were incubated for 24 h in 10 mL of Luria/Miller medium (LB medium; Carl Roth, Germany) at 37 °C. After that, 10^5^–10^6^ bacteria of each strain in 1 mL medium were added to every well plate having a scaffold in it and incubated for 24 h at 37 °C. After the specified time of incubation, Alamar Blue (Invitrogen, Schwerte, Germany) was added in order to analyze the metabolic activity of bacteria. Consequently, absorbance was measured at two different wavelengths (570 and 600 nm), and the reduction of Alamar Blue was determined by following the manufacturer’s protocol.

#### 2.5.7. In Vitro Cytocompatibility

In vitro cytocompatibility of the scaffolds was evaluated by using the osteosarcoma cell line MG-63 (Sigma Aldrich, Taufkirchen, Germany). Dulbecco’s Modified Eagle Medium (DMEM, Gibco, Thermo Fisher Scientific, Schwerte, Germany) containing 10 vol% fetal bovine serum (Sigma Aldrich, Taufkirchen, Germany) and 1% (*v*/*v*) penicillin/streptomycin (Sigma Aldrich, Taufkirchen, Germany) media supplements was used to culture the MG-63 cells. Prior to the cell seeding, all samples were UV-sterilized for 2 h. The 3D-printed scaffolds before and after coatings were placed in 24-well plates, and 10^5^ cells were directly seeded on the surfaces of the scaffolds and incubated at 37 °C for 1 and 3 days in order to evaluate initial cell attachment and cell proliferation behavior. After the specified time intervals, the scaffolds were transferred into a new well plate, and cell viability was assessed by using water-soluble tetrazolium salt (WST-8 assay, Sigma Aldrich). The reason to change the well plate before cell viability assessment was to avoid interference from cells that grow on the bottom of the well plate. A 1 vol% of WST-8 solution in DMEM was prepared and added into each type of scaffold. After 4 h of incubation with WST-8, optical absorbance was recorded at 450 nm.

For the fluorescence microscopic analysis, cells were stained by using Vybrant™ DyeCycle™ Violet Stain (ThermoFisher, Schwerte, Germany) and DAPI (4′,6-diamidino-2-phenylindole) (ThermoFisher, Schwerte, Germany). Vybrant™ DyeCycle™ Violet Stain was prepared in DMEM at a concentration of 4 µL/mL, whereas 1 µL/mL DAPI (4′,6-diamidino-2-phenylindole) was prepared in PBS solution. Before addition of DAPI, cells were fixed by using a fixing solution composed of 1 mM EGTA (Ethylene glycol tetra-acetic acid, Merck, Darmstadt, Germany), 4% (*w*/*v*) polyethyleneglycol, 3.7% (*w*/*v*) paraformaldehyde (all Sigma Aldrich, Taufkirchen, Germany) and 0.1 M PIPES (Piperazine-N, N′-bis (2-ethanesulfonic acid), Merck, Darmstadt, Germany), dissolved in PBS.

Fluorescence images were taken by using a microscope (Axio Scope A1, Carl Zeiss Microimaging GmbH, Jena, Germany). The samples were irradiated by their stimulating wavelength, and two images per magnification and sample were taken and combined into a two-colored image. Both 10× and 20× magnification images were taken in order to evaluate the morphology and proliferation of cells.

### 2.6. Statistical Analysis

All experiments were performed three times, and the results are presented as mean values and standard deviations (SDs). One way-ANOVA test was then applied to evaluate the statistical significance of biological studies, with *p* < 0.05 (*) considered statistically significant.

## 3. Results and Discussion

### 3.1. Surface Morphology, BG Distribution and Porosity

**Figure 1:**
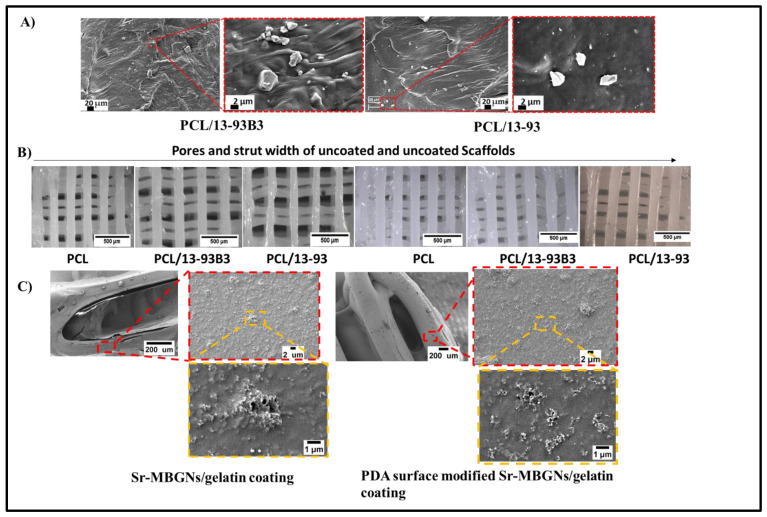
Surface morphology of (**A**) cross-section of filaments via SEM. (**B**) light microscopy images of scaffolds before and after coating. (**C**) SEM images of coated scaffolds at different magnifications highlighted in red and yellow dotted lines.

Scanning electron microscopy of the cross-section of PCL/BG filaments indicated a fairly homogenous dispersion of BG particles with some agglomeration throughout the PCL matrix (Figure 1A). Most of the BG particles were partially embedded in the matrix because the melted PCL during the composite filament fabrication covered the BG particles. BG particles have hydrophilic characteristics, whereas PCL is hydrophobic, which leads to incompatible mixing and some agglomeration of BG particles into the PCL matrix [32].

After fabrication of the scaffolds, they were first observed under a light optical microscope to evaluate the pore size and strut width. According to Distler et al. [20], better mechanical performance could be achieved with pore sizes of 750 μm. Moreover, different scaffold designs with controlled porosity could lead to a better stress distribution in comparison to the scaffolds fabricated in our study. The pore size and the strut width were calculated using the software ImageJ. It can be observed in Figure 1B that the pore size and strut width of the scaffolds varied with the addition of BG particles. The BG particle size has a great influence on the efficiency of printing. Larger BG particle sizes can lead to thinner struts and larger pores [33]. In Table 2, differences in pore size and strut width can be observed. The porosity of coated and non-coated porous scaffolds with a height of 3 mm and a diameter of 10 mm was also evaluated (Table 3). Compared to the pure PCL scaffolds, an increase in porosity could be observed in the BG composite scaffolds. This may have been due to the addition of BG particles into the polymer matrix, which influenced the printing of the scaffolds. After coating, the pore size was slightly reduced because of the coating layer on the surface of every strut.

SEM images of the scaffolds after coating showed that in the case of Sr-MBGNs/gelatin coating (without PDA modification), a very thin layer of coating material was present on the surface of the scaffolds. However, some cracks could be observed as well (Figure 1C), which might have been because of the weak adhesion of coating material onto the surface of hydrophobic PCL. The Sr-MBGNs/gelatin coating was more properly and homogeneously attached to the scaffold’s surface after PDA coating, forming a more homogeneous and crack-free coating layer. It has been reported in the literature that after the modification of a material with PDA, a thin layer of PDA acts as a secondary platform for binding functional molecules (e.g., drugs, nanoparticles, and proteins) to the surface [34]. This effect of a PDA layer was manifested here in the good surface adhesion of the coatings.

### 3.2. Contact Angle Measurement

**Figure 2:**
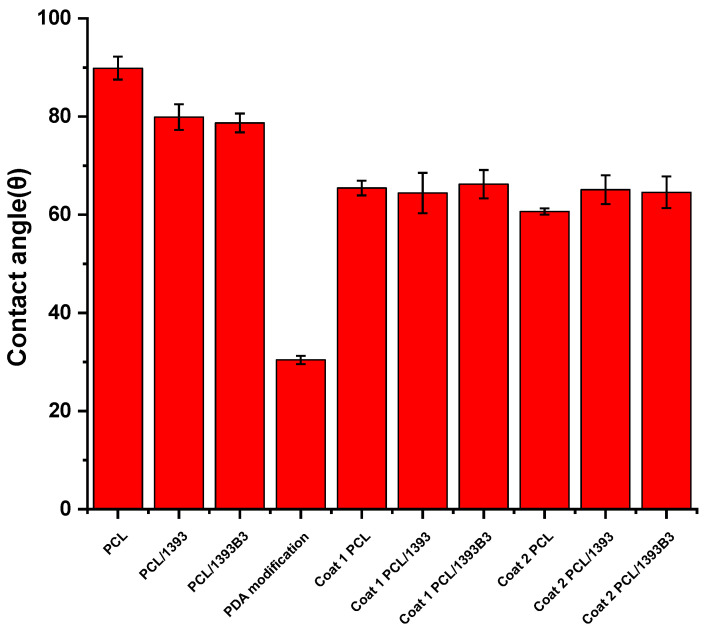
Comparison of contact angles of scaffolds before and after coatings.

Contact angle measurements of all coated and uncoated scaffolds were performed in order to evaluate the effect of modification and coating on the surface properties of the scaffolds. The contact angle before PDA surface modification seemed to be very high; Figure 2 confirms that the non-modified scaffolds had higher values of contact angle. PCL had a contact angle of 90 ± 2°, which was very close to the value given in the literature [35]. As discussed before, PCL has a hydrophobic nature. Chakrapani et al. [35] reported that pure PCL samples had a contact angle of 128°. In the case of Xia et al. [36], PCL had a contact angle of 112.98°. However, it was observed that the addition of BG increased the surface roughness and hydrophilicity, which may have caused the decrease in contact angle values. Figure 2 shows that the PCL/BG samples had lower contact angles compared to those of the pure PCL samples. PCL/13-93 composites showed a contact angle of 80 ± 3°, whereas PCL/13-93B3 composites exhibited a contact angle of 79 ± 2°. This can be explained by the influence of the hydrophilic BG particles added into the PCL matrix. The contact angle was strongly influenced after PDA surface modification. After 2 h of immersion, the contact angle was about 30.4 ± 0.8°. These values were much lower than those of the non-modified samples. The lower contact angle confirmed the enhanced hydrophilic properties of the surface, which were likely due to the catecholamine groups (N-H and O-H) of PDA [37]. The literature has shown that a suitable contact angle for cell culture is in the range of 5° to 40° [25]; therefore, the present values (30.4 ± 0.8°) were considered to be in a suitable range for enabling cell attachment.

The contact angle measurement was carried out also on samples coated with Sr-MBGNs/gelatin. Optical images of all coated samples in the experiment were taken. After comparing the contact angles of the non-coated samples, a clear difference between samples could be observed, which was due to the hydrophilic properties of gelatin. PDA-coated samples had a lower contact angle than the samples only coated with the gelatin solution. Gelatin-coated PCL, PCL/13-93 and PCL/13-93B3 had contact angles of 64 ± 1°, 65 ± 4° and 66 ± 3°, respectively, whereas after PDA modification, gelatin-coated PCL, PCL/13-93 and PCL/13-93B3 had contact angles of 60.6 ± 0.6°, 65 ± 3° and 65 ± 3°, respectively. The results demonstrate that the hydrophilicity of scaffolds improves after PDA surface modification [38]; therefore, the adhesion of the coating also improves.

### 3.3. In Vitro Degradation

**Figure 3:**
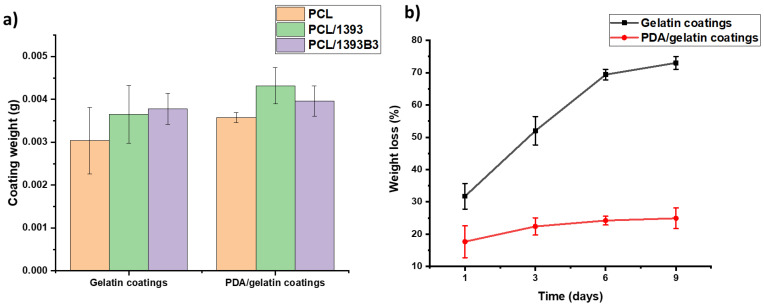
In vitro degradation; (**a**) weight of coatings; (**b**) degradation of different coated scaffolds soaked in DPBS solution for 9 days.

With surface modification and coating techniques, degradation time can be improved and tailored for a specific application. For this purpose, the weight of each scaffold was measured before and after coating. It can be observed in Figure 3a that PCL scaffolds had the lowest coated weight (3.04 ± 0.77 mg). However, after surface modification, the coated weight was improved, i.e., to 3.58 ± 0.12 mg. This may have happened because of the polydopamine coating, which reinforces the hydrophilic properties of PCL. On the other hand, the composite scaffolds of PCL/13-93 and PCL/13-93 B3 had average coated weights of 3.65 ± 0.68 mg and 3.78 ± 0.37 mg, respectively, whereas after PDA surface modification, the PCL/13-93B3 coated weight was 3.004 ± 0.004 g and that of PCL/1393 was 4.32 ± 0.43 mg. Thus, the surface modification of scaffolds with PDA enhanced the adhesion properties of the samples, which resulted in the higher coated weights of the scaffolds. Overall, PDA-modified scaffolds showed higher coated weights among all scaffolds, which proved the effectiveness of the surface modification. Hence, surface modification with PDA was confirmed as an important strategy to improve the effective coating of biomaterials onto the surfaces of PCL-based scaffolds [34].

The other important evaluation after confirmation of the presence of a homogenous coating is the determination of the degradation time of the coating. For this purpose, scaffolds were immersed in DPBS solution for about 2 weeks. Their weights, heights and diameters were measured before immersion and right after they were taken out of solution. Scaffolds coated with Sr-MBGNs/gelatin exhibited a faster degradation rate with much higher weight loss as compared to the scaffolds modified with PDA and coated with Sr-MBGNs/gelatin. Gelatin-coated scaffolds had 30% ± 4% weight loss after only 1 day and 73% ± 2% weight loss after 9 days, whereas PDA-and gelatin-coated scaffolds only had 18% ± 5% weight loss after 24 h and 25% ± 3% weight loss after 9 days. As observed in the SEM images of the coatings (Figure 1C), the Sr-MBGNs/gelatin coating exhibited numerous cracks as compared to the PDA-modified coating, which also demonstrated the positive effect of PDA-coated scaffolds. Additionally, genipin cross-linking is known to enhance the degradation properties of biopolymers for tissue engineering [39]. Degradation of different coated scaffolds soaked in DPBS solution for 9 days is described in Figure 3b.

### 3.4. Mechanical Properties

**Figure 4:**
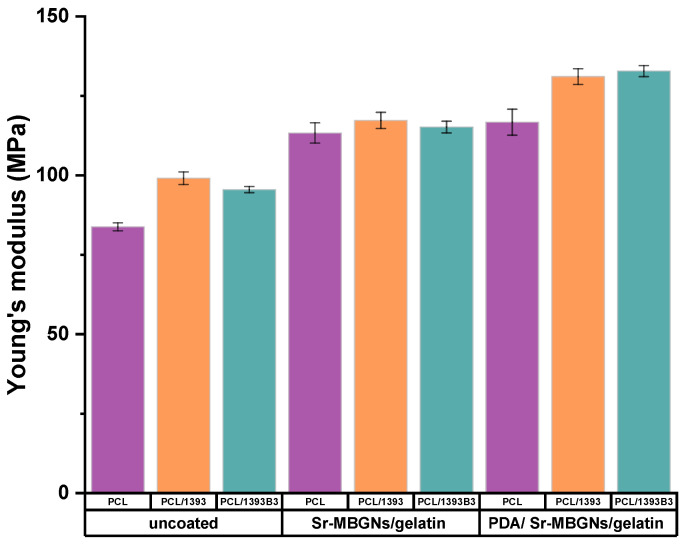
Young’s modulus of scaffolds. The data are represented as mean ± standard deviation (SD: n = 3).

Figure 4 depicts the compressive modulus (Young’s modulus) of 3D-printed coated and uncoated scaffolds. It was observed that PCL had a Young’s modulus of 84 ± 1 MPa. However, after the inclusion of BG particles, the Young’s modulus of the scaffolds increased (Figure 4). The increase in the Young’s modulus of composite scaffolds was expected, as in previous studies (Korpela et al. [40]). Moreover, Jiang et al. [41] also explained that the inclusion of BG particles with <50 μm particle size into PCL matrix caused an increase in the Young’s modulus of the scaffolds. It was observed that the Young’s modulus of Sr-MBGNs/gelatin-coated scaffolds was considerably higher compared to that of non-coated PCL/BG scaffolds. It was previously reported that the exchange of calcium with strontium causes an expansion of the silicate-glass network [42,43], which controls the crystallization of the orthophosphate phase [43]. It was also reported that size as well as crystalline structure within a composite system may affect the interfacial adhesion and, therefore, affect the mechanical properties of the composite [44,45]. The results are also related to the report of Gerhardt et al. [46], who explained that the glass phase composition significantly influenced the mechanical strength of scaffolds, even though the mechanism behind the interfacial interaction between 13-93, 13-93B3 and Sr-MBGNs with PCL is unclear. Moreover, gelatin cross-linked with genipin may improve the mechanical properties of coated scaffolds [29,39]. In addition to that, Chi et al. [47] observed that PDA modification can also increase the mechanical properties of the scaffolds. In that study, it was observed that the compressive modulus of the 3D-printed polylactic acid scaffolds significantly increased due to the PDA/ hydroxyapatite coatings.

### 3.5. In Vitro Bioactivity

**Figure 5:**
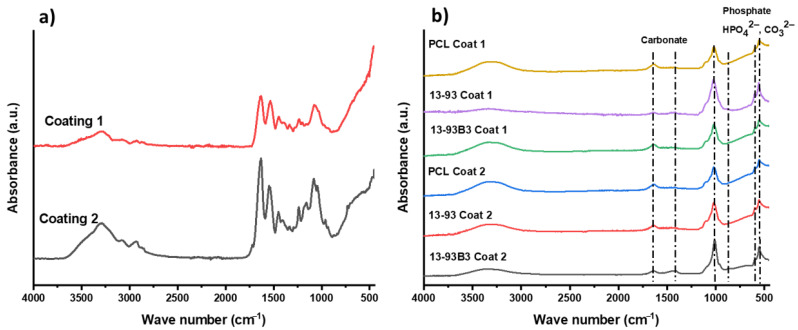
FTIR spectra of coated scaffolds before (**a**) and after (**b**) 7 days of immersion in SBF. The relevant bands are described in the text.

The study of hydroxyapatite (HA) formation on the surfaces of coated as well as non-coated scaffolds after immersion in SBF solution was performed in order to assess the bioactivity of the scaffolds. The HA formation was investigated by using FTIR and SEM. The results of FTIR measurements on the scaffolds before SBF immersion (0 day) and after immersion for 7 days (for coated scaffolds) are shown in Figure 5. FTIR spectra of non-coated scaffolds are not shown, as there were no clear hydroxyapatite peaks in the case of non-coated scaffolds because the melted PCL encapsulated the BG particles, and the hydroxyapatite layers formed on the surface were too thin to be detected. On the other hand, the FTIR spectrum (Figure 5b) of coated scaffolds after 7 days clearly showed two bands at 566 cm^−1^ and 603 cm^−1^, which confirmed the presence of P–O bending vibrations in crystalline HA [48]. Furthermore, the presence of stretching vibrations of C–O bonds was confirmed by the presence of a band at 873 cm^−1^ and dual broad bands at 1420–1450 cm^−1^, which illustrated that the HA layer was actually carbonated hydroxyapatite [48]. Additionally, the Sr-MBGNs in the gelatin coating material had high silicon and calcium content [49], which leads to HA layer creation on the surface of coated samples [50].

**Figure 6:**
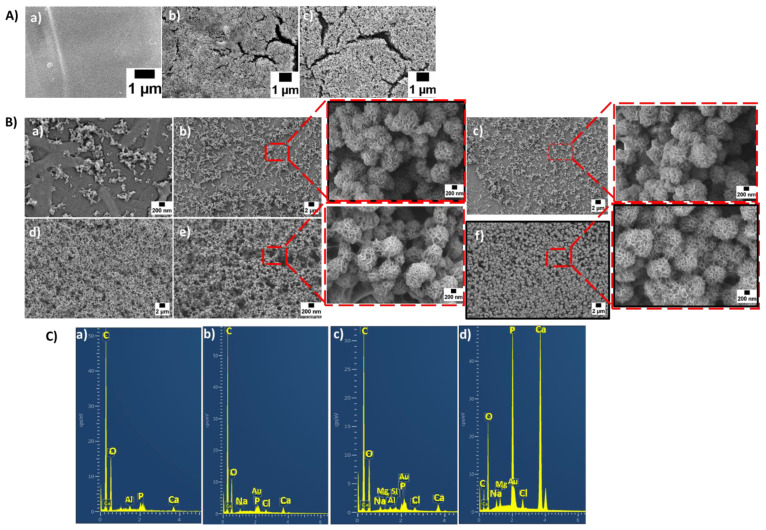
SEM images of uncoated scaffolds after immersion in SBF for 28 days (**A**): PCL (**a**), PCL/13-93 (**b**), PCL/13-93B3 (**c**). Coated scaffolds after immersion in SBF for 7 days at different magnifications (**B**): PCL Coat 1 (**a**), PCL/13-93 Coat 1 (**b**), PCL/13-93B3 Coat 1 (**c**), PCL Coat 2 (**d**), PCL/13-93 Coat 2 (**e**), PCL/13-93B3 Coat 2 (**f**). EDX spectra of uncoated and coated scaffolds (**C**): PCL/13-93 uncoated (**a**), PCL/13-93B3 uncoated (**b**), PCL/13-93B3 Coat 1 (**c**), PCL/13-93B3 Coat 2 (**d**).

In addition, Figure 6A–C show SEM images of uncoated and coated scaffolds as well as the respective EDX spectra of scaffolds after immersion in SBF solution. It was observed that in the case of PCL samples, no HA layer could be observed on their surfaces even after 28 days of immersion in SBF solution because there were no BG particles to exchange the ions for the development of an HA layer. It was observed that an HA layer began to appear after 3 days of immersion in the case of coated scaffolds, and after 7 days, it became quite homogeneous. A globular and cauliflower-like HA layer was clearly visible, especially at higher magnifications in Figure 6B. As PDA modification improved the adhesion of the coating, a more homogeneous HA layer formation could be observed. The existence of such a high amount of calcium phosphate on Sr-MBGNs/gelatin- as well as on PDA- modified Sr-MBGNs/gelatin-coated samples proved conclusively that the coatings had a strong influence on the bioactivity of the scaffolds. The surface morphologies of uncoated scaffolds are also shown for comparison. It can be observed that in non-coated PCL/13-93 and PCL/13-93B3 scaffolds after the early stage of SBF immersion (7 days), a very low formation of HA was apparent on the surface. However, with the increase in the immersion time to 14 days, the HA layer on the uncoated scaffolds increased. After 28 days, a more extensive layer of HA could be observed (Figure 6A). This proves that the bioactivity of the BGs influenced the polymer material positively. As most of the BG particles were embedded into the polymer matrix, there was no exposure of BG particles to SBF, and the exchange of ions was difficult, which resulted in a very thin layer and extended the period of time required for the HA formation as mentioned above.

An EDX analysis of scaffolds was also performed to evaluate the calcium and phosphate ratio of the apatite layer formed onto their surfaces. The presence of Ca and P peaks confirmed that an HA layer had indeed formed on the surfaces of the samples. Stoichiometric HA layers should have a Ca/P ratio of 1.67. The EDX results for the coated scaffolds after 7 days of immersion in SBF solution are shown in Figure 6(Cc,Cd). In the case of coating 1, all scaffolds showed similar EDX spectra; therefore, only one spectral band is shown in Figure 6C. This was also the case with coating 2. The EDX spectrum of PDA/gelatin-Sr-MBGNs-coated scaffold exhibited much sharper peaks and higher intensities of calcium and phosphate. This result indicates that the hydroxyapatite layer had formed to a large extent after 7 days of immersion in SBF solution. It can be observed from the EDX evaluation that the PDA-modified Sr-MBGNs/gelatin coating is indeed an attractive coating technique to increase the bioactivity of scaffolds.

### 3.6. Anti-Bacterial Study

**Figure 7:**
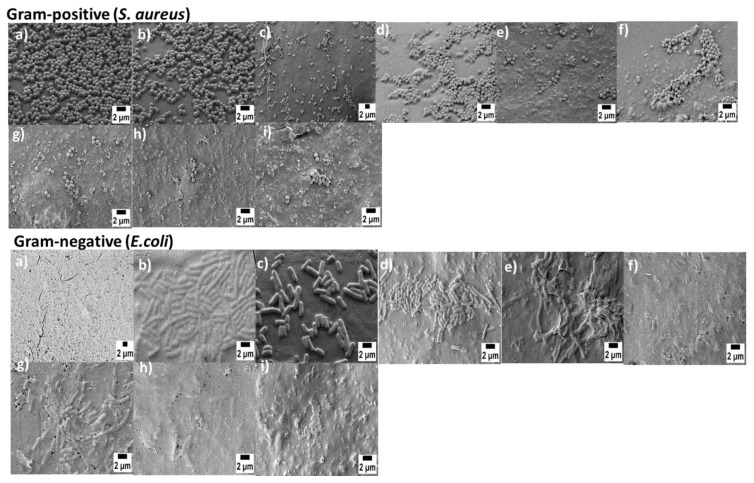
SEM images showing antibacterial effect on Gram-positive *S. aureus* (**top**): PCL (**a**), PCL/13-93 (**b**), PCL/13-93B3 (**c**), PCL Coat 1 (**d**), PCL/13-93 Coat 1 (**e**), PCL/13-93B3 Coat 1 (**f**), PCL Coat 2 (**g**), PCL/13-93 Coat 2 (**h**), PCL/13-93B3 Coat 2 (**i**), and on Gram-negative *E. coli* bacteria (**bottom**): PCL (**a**), PCL/13-93 (**b**), PCL/13-93B3 (**c**), PCL Coat 1 (**d**), PCL/13-93 Coat 1 (**e**), PCL/13-93B3 Coat 1 (**f**), PCL Coat 2 (**g**), PCL/13-93 Coat 2 (**h**), PCL/13-93B3 Coat 2 (**i**).

**Figure 8:**
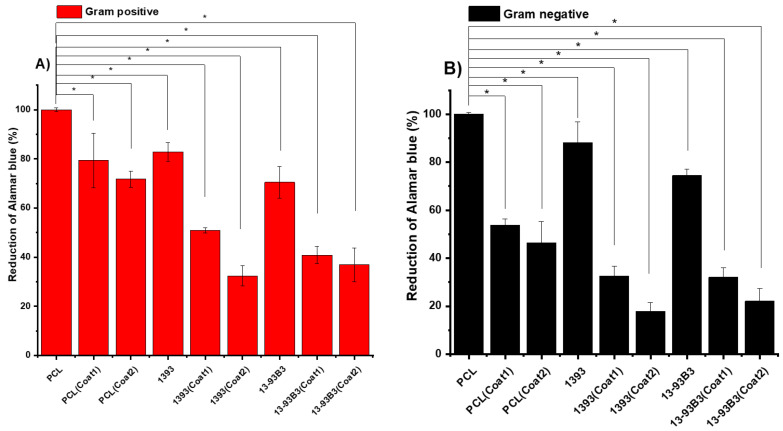
Reduction of Alamar blue after 24 h: (**A**) Gram-positive (*S. aureus*) and (**B**) Gram-negative (*E. coli*) bacteria, with *p* < 0.05 (*) considered statistically significant.

The anti-bacterial properties of scaffolds were investigated on uncoated and coated scaffolds against Gram-positive and Gram-negative bacteria. Bacterial attachment was observed with SEM (Figure 7). To confirm which components in the composite scaffolds played the key role in antibacterial activities, we set pure PCL scaffolds as the control group for both strains. The graphs were plotted to represent the percentage of viable bacterial cells in the case of both Gram-positive and Gram-negative bacterial strains (Figure 8). As the results show, only the pure PCL scaffolds were unable to inhibit both strains of bacterial growth. The reduction in the Alamar blue absorbance was directly related to the relative number of bacteria. Bare PCL scaffolds had the highest value of Alamar Blue reduction (≅70%) against Gram-negative bacteria. Interestingly, percentage viability was reduced in the case of PCL/BG scaffolds (about ≅37% for PCL/1393 and 31% for PCL/1393B3). Coated scaffolds incorporating Sr-MBGNs/gelatin demonstrated potent antibacterial activity against bacteria. After PDA surface modification, the percentage viability was reduced even more. The exact mechanisms for the antibacterial effect of BGs have not been completely elucidated [51]. The presence of different ions such as calcium [51], phosphate [51] and strontium [52] and an increased value of pH are commonly considered as factors causing an antibacterial effect [53]. Moreover, Kao et al. [25] revealed that PDA-PLA showed a higher bacterial mortality rate as compared to neat PLA. This finding indicates that PDA could increase the antibacterial effect in the coating layer. Additionally, surface-modified PDA scaffolds were also capable of decreasing protein adhesion; as a consequence, bacterial binding could be retarded during an adhesion experiment [23]. The results were also confirmed by SEM (Figure 7). Previously, Sureshkumar et al. [54] prepared a multilayer coating with multi-metal nanoparticles onto the surface of a polymer film by first modifying the film with the extraordinarily reductive and adhesive self-polymerized polydopamine. They observed that this hybrid film exhibited antibacterial properties. It was also observed that combining gelatin with Sr-doped BG reduced the expression of the cytokine IL-6 gene, a proinflammatory biomolecule [55], leading to better antibacterial activity followed by enhanced bone tissue regeneration. Baheiraei et al. [55] reported that substitution with Sr had antibacterial properties against *S. aureus* and *E. coli*. Therefore, as a conclusion of our experimental outcomes and discussion combined with the previous literature, the improved antibacterial properties of coated scaffolds can be ascribed primarily to the presence of Sr-MBGNs that are directly attached to the polydopamine layer on the surface of the scaffolds.

### 3.7. In Vitro Cytocompatibility

**Figure 9:**
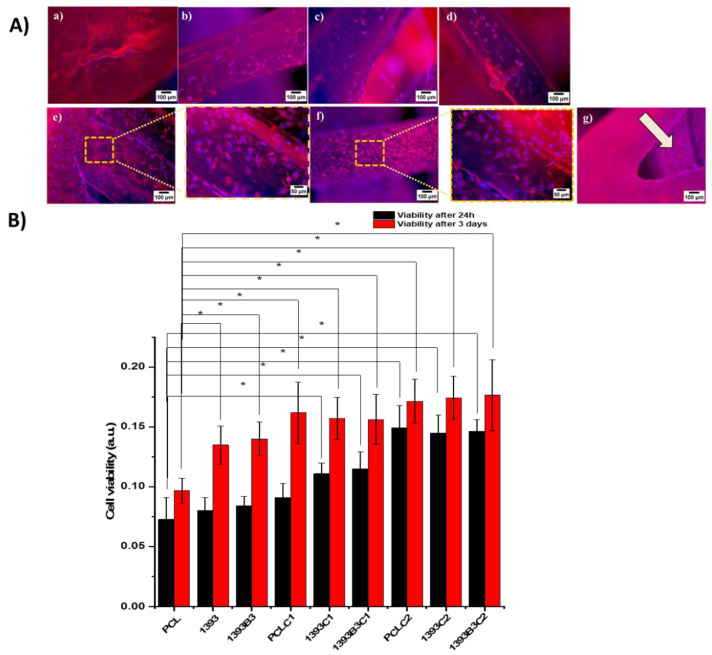
Results of direct cell analysis of coated and uncoated scaffolds with osteoblast-like cell line MG-63. (**A**) Fluorescence images of uncoated PCL (**a**), PCL/13-93 (**b**), PCL/13-93B3 (**c**), and coated scaffolds PCL (**d**), PCL/13-93 (**e**), PCL/13-93B3 (**f**), and cell attachment on the inner struts (**g**). (**B**) Cell viability of all scaffolds after 24 h and 3 days, with *p* < 0.05 (*) considered statistically significant.

The viability and spreading behavior of MG-63 cells was studied on all coated and non-coated scaffolds. The viability of cells on the scaffolds was compared relative to 1 day of incubation on pure PCL scaffolds as a control. Figure 9A presents the cell attachment on uncoated scaffolds, and it can be observed that only a few cells had the chance to attach on the surface of the PCL scaffolds. Moreover, the morphology of these cells revealed a round shape, whereas the cell proliferation in the case of the PCL/BG composite scaffolds was significantly higher compared to that of PCL scaffolds. As expected, the incorporation of bioactive glass particles into the polymer matrix stimulated cell proliferation. Therefore, more grown cells were present on the top of the composite scaffolds. After 1 and 3 days, the OD percentages of the composite scaffolds were significantly higher than those of the neat polymer PCL scaffold. However, the number of cells still could be considered low, which could be ascribed to the fact that most of the BG particles were encapsulated in PCL during the fused deposition process, and hydrophobic PCL had no active sites that would encourage cellular attachment. These properties can hinder cell adhesion and migration [56,57]. This was precisely the reason for exploring the development of bioactive coatings in this study. Thus, we introduced hydrophilicity by coating the PCL and PCL-BG composite scaffolds using PDA surface modification in order to improve cell attachment and proliferation. Previously, Jo et al. [58] reported that plotted 3D PCL scaffolds with polydopamine surface modification had enhanced cell proliferation into the scaffolds. Figure 9A compares the morphology of the osteoblast-like cells attached onto coated scaffolds at different magnifications. It was observed that both types of coated scaffolds could provide suitable sites for cell attachment and adhesion. However, scaffolds with PDA surface-modified Sr-MBGNs/gelatin coatings showed high confluency of osteoblast-like cells even at the inner struts. In the case of un-modified coated scaffolds, the attachment of the coating material to the hydrophobic PCL was poor, which caused thin and non-homogeneous coatings that could also be observed in SEM images. According to the literature, surface modification with PDA can create covalent and noncovalent interactions; for example, it may interact via hydrogen bonding or π-π and electrostatic interactions [59]. This effect might be a reason for the good adhesion of the coating material leading to more cell attachment because of the presence of bioactive nanoparticles in gelatin. These considerable differences observed indicate that the existence of MBGNs promotes cell growth. Moreover, the surface modification with PDA prevented protein denaturation and, therefore, enhanced cell adhesion [59]. Additionally, the OD (Figure 9B) values of osteoblastic-like cells on PDA surface-modified Sr-MBGNs/gelatin-coated scaffolds indicated a significant number of living cells compared to those without PDA modification. The results thus confirmed that the scaffolds are suitable for interaction with osteoblast-like cells. Hence, based on the results of the proliferation test, we considered that the 3D-printed and coated scaffolds developed in this study fulfilled the basic requirement for bone tissue engineering, namely providing a suitable 3D environment for cell proliferation.

## 4. Conclusions

In this study, we observed that mussel-inspired PDA surface modification can improve the quality of hydrophobic PCL-based composite scaffolds. The hydrophilicity induced on the surfaces of the scaffolds improved the quality and adhesion of the coating. As a consequence, the homogenous coatings of the scaffolds led to significant improvement in the physicochemical and mechanical properties as well as the in vitro bioactivity. Moreover, the PDA-modified Sr-MBGNs/gelatin coating was shown to significantly promote cell attachment and proliferation as compared to unmodified scaffolds. Furthermore, surface enrichment of scaffolds after coating demonstrated better antibacterial properties. Therefore, we conclude that this simple method of surface modification of PCL composite scaffolds with PDA and Sr-MBGNs/gelatin will lead to promising bio-inspired scaffolds with suitable cell proliferation capability and effective antibacterial properties for bone tissue engineering applications.

## Figures and Tables

**Table 1:** Nominal composition of bioactive glasses in wt%.

Composition (wt%)	13-93	13-93B3
Na_2_O	5.5	5.5
K_2_O	11.1	11.1
MgO	4.6	4.6
CaO	18.5	18.5
SiO_2_	56.6	0
P_2_O_4_	3.7	3.7
B_2_O_3_	0	56.6

**Table 2:** Strut width and pore size of uncoated scaffolds (ImageJ).

Sample	Strut Width (μm)	Pore Size (μm)
PCL	293 ± 15	284 ± 8
PCL/13-93	267 ± 19	299 ± 40
PCL/13-93B3	214 ± 19	350 ± 37

**Table 3:** Porosity values of PCL and PCL/BG composite scaffolds before and after coating.

Samples	Before Coating (%)	After Sr-MBGNs/Gelatin Coating (%)	After PDA/Sr-MBGNs/Gelatin Coating (%)
PCL	72 ± 1	72 ± 1	71 ± 1
PCL/13-93	78 ± 2	76 ± 1	77.3 ± 0.6
PCL/13-93B3	79.3 ± 0.8	77.8 ± 0.7	77.2 ± 0.6

## Data Availability

Not applicable.
